# High Fat Diet Induced Developmental Defects in the Mouse: Oocyte Meiotic Aneuploidy and Fetal Growth Retardation/Brain Defects

**DOI:** 10.1371/journal.pone.0049217

**Published:** 2012-11-12

**Authors:** Kerri M. Luzzo, Qiang Wang, Scott H. Purcell, Maggie Chi, Patricia T. Jimenez, Natalia Grindler, Tim Schedl, Kelle H. Moley

**Affiliations:** 1 Washington University School of Medicine, Department of Obstetrics and Gynecology, St. Louis, Missouri, United States of America; 2 State Key Laboratory of Reproductive Medicine, Nanjing Medical University, Nanjing, China; 3 Washington University School of Medicine, Department of Genetics, St. Louis, Missouri, United States of America; McGill University, Canada

## Abstract

**Background:**

Maternal obesity is associated with poor outcomes across the reproductive spectrum including infertility, increased time to pregnancy, early pregnancy loss, fetal loss, congenital abnormalities and neonatal conditions. Furthermore, the proportion of reproductive-aged woman that are obese in the population is increasing sharply. From current studies it is not clear if the origin of the reproductive complications is attributable to problems that arise in the oocyte or the uterine environment.

**Methodology/Principal Findings:**

We examined the developmental basis of the reproductive phenotypes in obese animals by employing a high fat diet mouse model of obesity. We analyzed very early embryonic and fetal phenotypes, which can be parsed into three abnormal developmental processes that occur in obese mothers. The first is oocyte meiotic aneuploidy that then leads to early embryonic loss. The second is an abnormal process distinct from meiotic aneuploidy that also leads to early embryonic loss. The third is fetal growth retardation and brain developmental abnormalities, which based on embryo transfer experiments are not due to the obese uterine environment but instead must be from a defect that arises prior to the blastocyst stage.

**Conclusions/Significance:**

Our results suggest that reproductive complications in obese females are, at least in part, from oocyte maternal effects. This conclusion is consistent with IVF studies where the increased pregnancy failure rate in obese women returns to the normal rate if donor oocytes are used instead of autologous oocytes. We postulate that preconceptional weight gain adversely affects pregnancy outcomes and fetal development. In light of our findings, preconceptional counseling may be indicated as the preferable, earlier target for intervention in obese women desiring pregnancy and healthy outcomes.

## Introduction

Approximately 60% of women desiring pregnancy in the US are overweight by the BMI criterion, with incidence rising exponentially over the past 15 yrs [1]. Overweight and obese women experience poorer reproductive outcomes than normal weight women, including increased rates of infertility and pregnancy loss, as well as fetal and neonatal problems such as developmental delay and neurological deficits, respectively [2]–[7]. In addition, children born to obese mothers are more likely to acquire childhood obesity. The developmental problems could arise from compromised oocyte or embryo development, impaired uterine environment, or a combination of these factors. Importantly, a recent study of IVF pregnancies found that the increased pregnancy failure rate in obese women reverses back to a normal rate if donor oocytes are used instead of autologous oocytes, indicating that oocyte quality in obese women is compromised and contributes significantly to pregnancy complications [8].

The impact of abnormal maternal metabolic environment on oocyte quality and pregnancy outcomes was initially seen in diabetic mouse models. Female mice with models of type 1 diabetes produce oocytes that are smaller show impaired maturation and increased granulosa cell apoptosis [9]–[12] and display poor reproductive outcomes including growth restriction and congenital anomalies [13], [14]. The growth restriction and congenital abnormalities were found to be the result of an oocyte maternal effect rather than a diabetic uterine environmental effect as transfer of one-cell zygotes derived from diabetic mothers to control non-diabetic mothers failed to rescue the developmental defects. Likewise, high fat diet mouse models of obesity display similar negative impacts on the oocyte, the embryo, and pregnancy outcomes. Oocytes from obese mice are smaller, show delayed meiotic maturation, have increased follicular apoptosis [15] and their offspring exhibit embryonic developmental defects [16], [17] and growth retardation [15].

One proposed mechanism for compromised oocyte quality and poor reproductive outcomes in obese females includes altered mitochondrial activity at the oocyte stage. Abnormal mitochondria structure and function in oocytes from type 1 diabetic mice are associated with poor fertilization rates and abnormal embryo development [18]. It is apparent that both obesity and diabetes influence mitochondrial activity. In obese mice, uneven mitochondrial distribution and increased mitochondrial DNA copy number are correlated with impaired embryonic development from the zygote to blastocyst stage [19]. Oocytes from diabetic mice have increased abnormalities in mitochondria morphology, distribution, and mtDNA copy number [18]. Oocytes from diabetic mice also exhibit spindle defects and chromosome misalignment [18], which that can be associated with mitochondrial metabolism [20], [21], and changes in endocrine surroundings [21].

Here we sought to further investigate the developmental origins of reduced fertility in a mouse model of obesity, examining the oocyte, early embryo and whether the obese uterine environment is required for the observed fetal developmental abnormalities. We report that mice maintained on a high-fat diet produce oocytes with meiotic spindle and chromosome alignment abnormalities, which will lead to aneuploidy embryos. We find that transfer of embryos that developed normally to the blastocyst stage into non-obese dam fails to rescue the growth retardation in the resulting fetuses, many of which have brain abnormalities, demonstrating that the initiating defect occurred earlier than the blastocyst stage, possible in the oocyte. Our results suggest that reduced fertility in obese females is, at least in part, oocyte specific.

## Materials and Methods

### Animals and Diet

This study was carried out in strict accordance with the recommendations in the Guide for the Care and Use of Laboratory Animals of the National Institutes of Health. The protocol was approved by the IACUC accredited Animal Studies Committee of Washington University School of Medicine [Study # 20120051]. Female ICR mice [Taconic Laboratories, Hudson, NY] age 3 weeks were housed 5 per cage. Mice were fed either a high fat diet [HFD] [Diet 58R3; TestDiet, Richmond, IN] containing 36% fat, and 20% protein by nutritional content or an isocaloric control diet [PicoLab Mouse diet 20; LabDiet; Richmond, IN] containing 13% fat and 25% protein for a total of 4 weeks.

**Figure 1:**
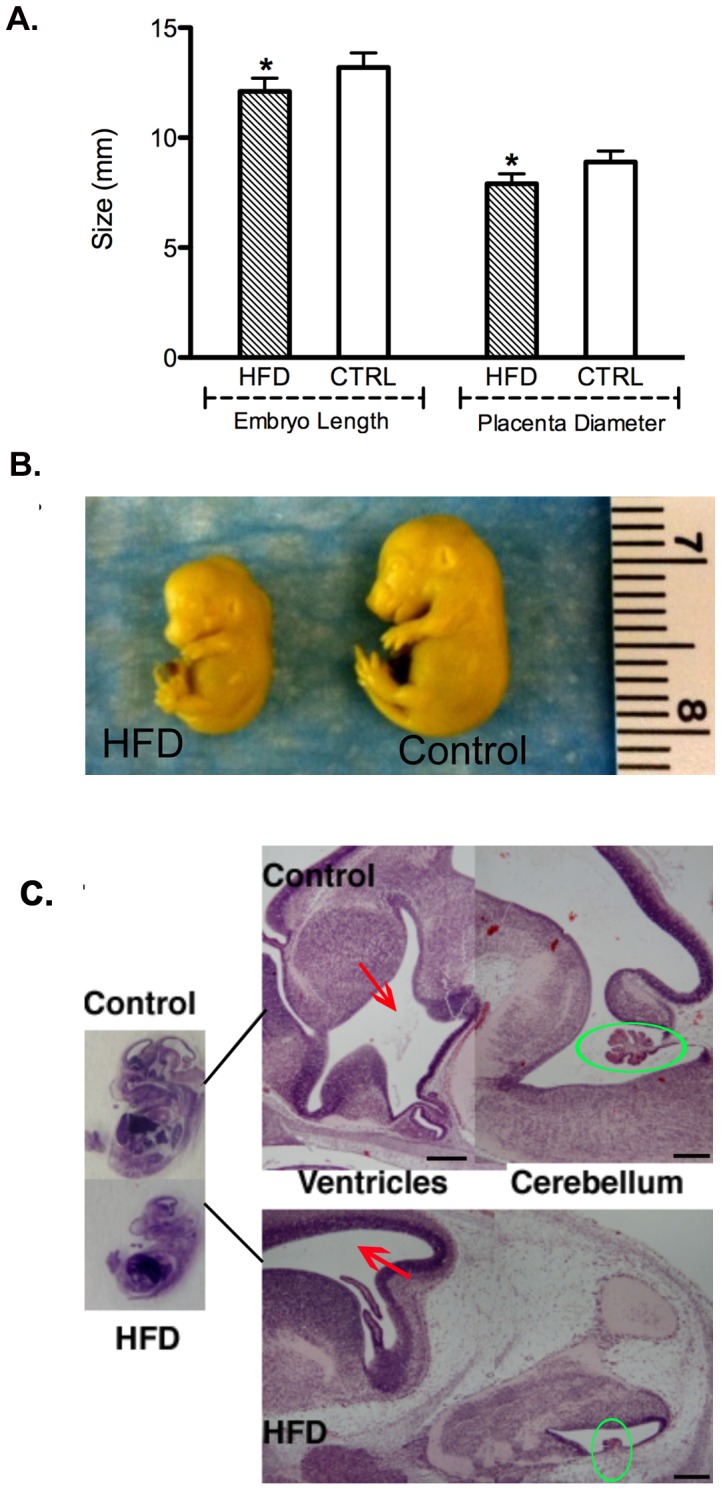
Fetal and placenta size and brain abnormalities. [A] Embryos from mothers on a high fat diet were significantly smaller than those from a control diet [12.1 mm vs 13.2 mm; *p<0.001]. Similarly, placentas from the HFD mothers were significantly smaller than placentas originating from control blastocysts [7.9 mm vs 8.9 mm; *p<0.001]. [B] d14.5 fetuses from HFD [left] and Control [right]; example shown for HFD illustrates the more extreme end of the spectrum of growth retarded fetuses. [C] H & E staining of control fetus and fetal brain vs HFD fetus and HFD fetal brain. All fetuses were from transferred blastocysts. Abnormal development of both the ventricles and the choroid plexus is seen in 40% of the HFD blastocyst-derived fetuses. Scale bars, 500 µm.

### Embryos

We transferred blastocysts from either control or HFD mice directly into pseudopregnant control recipients as previously described [14]. On embryonic day 14.5 mice were sacrificed, and embryos and placentas were collected and evaluated for length, weight, and external morphologic abnormalities. Embryos were then fixed in Bouin’s solution at room temperature for 24 h, embedded in paraffin, and serially sectioned at 5 µm. Sections were rehydrated and stained with hematoxylin-eosin and read by a pathologist blinded to the origin of the embryos. All major organs were examined. Major and minor malformations were noted, as well as any other developmental abnormalities. Implantation and pregnancy rates were recorded. Each experiment was run in triplicate for a total of 15 mice per group; we examined 53 embryos and placentas from the HFD group and 49 embryos and placentas from the control group.

**Figure 2:**
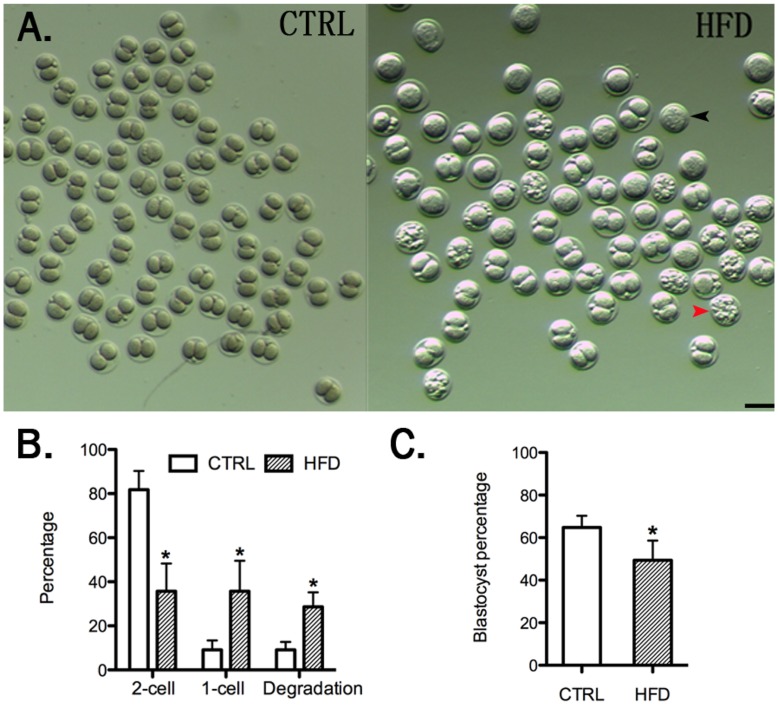
Embryos from HFD mice experience a higher frequency of degradation and delayed developmental progression. [A] Representative image of embryos collected from control and HFD mice at 46 h post-hCG. Red and black arrowheads indicate degraded and 1-cell embryos, respectively. Scale bar, 100 µm. [B] Quantification of 1-cell, 2-cell and degraded embryos from control and HFD mice [n = 110 control embryos from 6 mice and n = 184 HFD embryos from 9 mice, pooled from three replicates]. [C] Histogram showing the blastocyst formation rate of 2-cell embryos from control and HFD mice. [n = 90 for control and n = 65 for HFD pooled from three replicates]. Error bars indicate ± sd. *, P<0.05 vs. control.

### Pre-implantation Embryo Retrieval and Culture

Mice received an injection of 10 IU hCG 2 days after PMSG priming and were then mated overnight with males of proven fertility. Mating was confirmed by the presence of a vaginal plug. Two-cell embryos were obtained by flushing the oviducts 46 h post-hCG. For the *in vitro* culture study, the embryos were incubated for 48 h in KSOM [Millipore Specialty Media, Danvers, MA, USA] and the indicated culture conditions at 37°C in 5% CO_2,_ 5% O_2_ and 90% N_2_.

### Spindle and Chromosome Imaging

Mice from both diet groups were initially injected intraperitoneally with 10 IU of PMSG, followed by 5 IU of human chorionic gonadotropin [hCG] 48 hours later. Twelve to 14 hours later, mice were sacrificed, and cumulus enclosed oocytes were collected from the ampulla. Visualization of oocyte spindle and chromosome organization was conducted as previously described [18]. Each experiment was performed in triplicate. In total we evaluated 64 and 38 MII spindles for the HFD and control groups, respectively, and 67 and 40 for chromosome organization in the HFD and control groups, respectively.

### Mitochondria Ultrastructure and Morphology of Oocytes and Cumulus Cells

After 4 weeks on either the HFD or control diet, mice were injected with 10 IU of pregnant mare serum gonadotropin [PMSG] [Sigma, St Louis, MO] to promote superovulation. 48 hours, mice were sacrificed, the ovaries were collected, and germinal vesicle [GV] stage oocytes were obtained by manual rupturing of ovarian follicles. Oocytes were either maintained in the cumulus-enclosed state, or cumulus cells were removed via repetitive pipetting to obtain denuded oocytes. Oocytes with or without cumulus cells were processed for transmission electron microscopy for ultrastructural analysis as previously described [22]. Electron micrographs at 25,000×magnification were evaluated blindly. This experiment was repeated in triplicate; in total, we examined 230 mitochondria from 10 HFD oocytes from 5 mice and 299 mitochondria from 10 CTRL oocytes from 5 mice.

**Figure 3:**
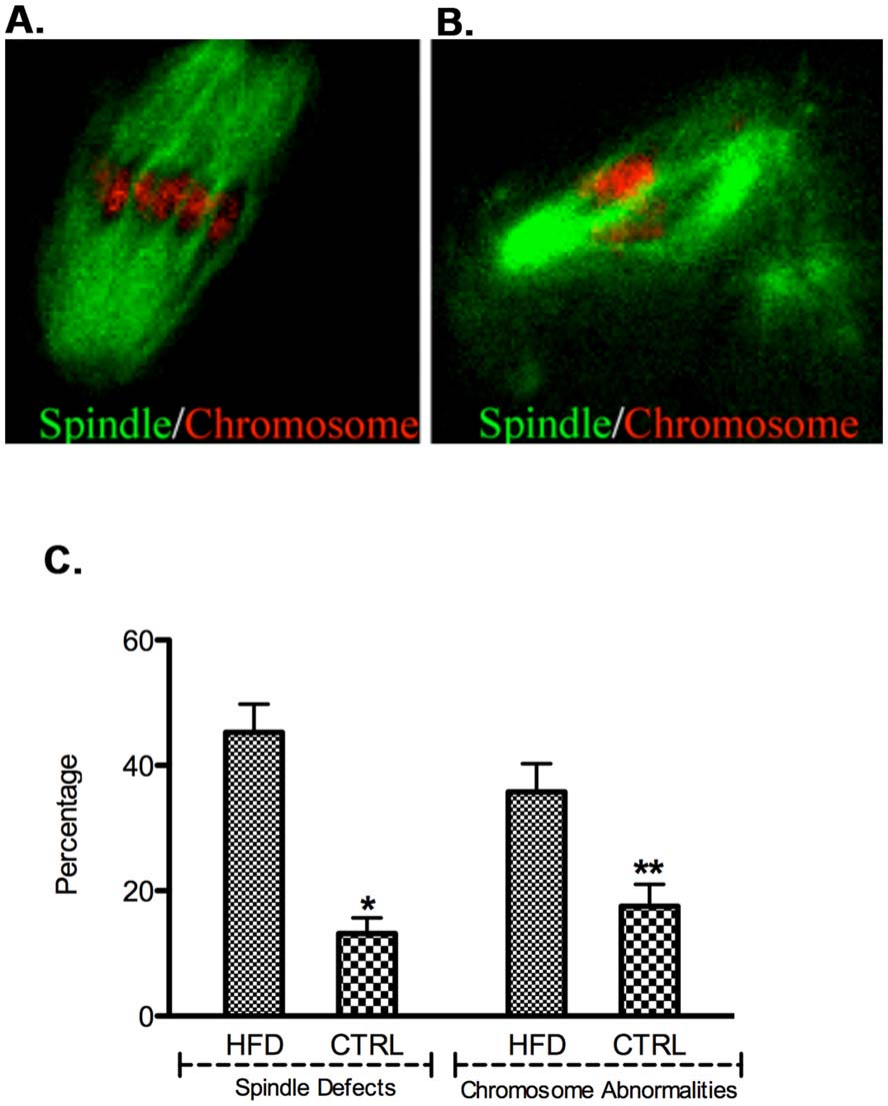
Oocyte MII spindles and chromosome alignment defects. [A] The MII oocytes from control mice display a typical barrel shaped spindle and organized chromosomes on the metaphase plate. Oocytes from HFD mice [B] revealed abnormal fragmented appearing spindles and clustered chromosomes. [C] Quantitative analysis revealed both spindle defects [45.3% vs 13.2%, *p = 0.0008] and chromosome misalignment [35.8% vs 17.5%, **p = 0.04] were increased in HFD fed mice compared to control mice. N = 146 control oocytes from 7 mice and n = 138 HFD oocytes from 7 mice were analyzed. Data are expressed as mean percentage ± SD from three independent experiments.

### Mitochondrial Function-metabolite Levels and Protein Expression

#### Mitochondrial Function

Denuded GV oocytes were obtained as described above and frozen on a glass slide by dipping in isopentane equilibrated with liquid nitrogen. After freeze-drying overnight under vacuum at −35**°**C, the oocytes were extracted in a nanoliter volume under oil. ATP and citrate levels were measured using an enzyme-linked assay as previously described [23]. This experiment was completed 3 times with at least 15 oocytes in each group for each experiment.

#### Immunofluorescence

Denuded GV oocytes were obtained as described above and prepared for immunofluorescence as described previously [24]. Fixed oocytes were incubated consecutively in 1∶500 polyclonal anti-DRP1 [Novus Biologicals [NB110-55288] Littleton, Colorado] for 1 hr, an appropriate fluorescein-tagged secondary antibody for 20 min, and To-Pro-3-iodide [Molecular Probes, Eugene, OR] for 20 min. All incubations were at room temperature in a humidified chamber, and the slides were washed three times in PBS/BSA between each incubation step and prior to mounting in VectaShield [Vector Labs, Burlingame, CA]. Samples were covered with cover slips, sealed, and examined with an Olympus laser-scanning confocal microscope. The experiment was completed three times with at least 15 oocytes for each experiment. One representative image for each group is shown.

#### Western Immunoblot

200 denuded GV oocytes from 10 mice were added directly to Laemmli sample buffer, boiled for 5 min, subjected to SDS-PAGE on 10% acrylamide gels, and transferred to nitrocellulose. Blots were processed according to standard Western blot procedures using polyclonal anti-PGC-1α[EMB Millipore, Billerica, MA, AB3242] or 1∶500 polyclonal anti-DRP1 primary antibodies, anti-rabbit secondary, and SuperSignal West Dura ECL kit [Pierce Chemical Co., Rockford, IL]. All experiments were done three times and two representative immunoblots are shown.

### Determination of mtDNA Copy Number by Quantitative Real-time PCR

The mtDNA extraction and quantitative real-time PCR procedure were done as previously described [25]. Linear regression analysis of all standard curves for samples with copy number between 10^2^ and 10^6^ showed a correlation coefficient higher than 0.98. All measurements were performed in triplicate.

### Statistical Analysis

Data were analyzed using Student’s t test or Chi squared where appropriate. A P value of <0.05 was considered significant. For analysis of the equality of continuous distribution of fetal sizes and differences between mothers, a Kolmogorov-Smirnov test was used.

**Figure 4:**
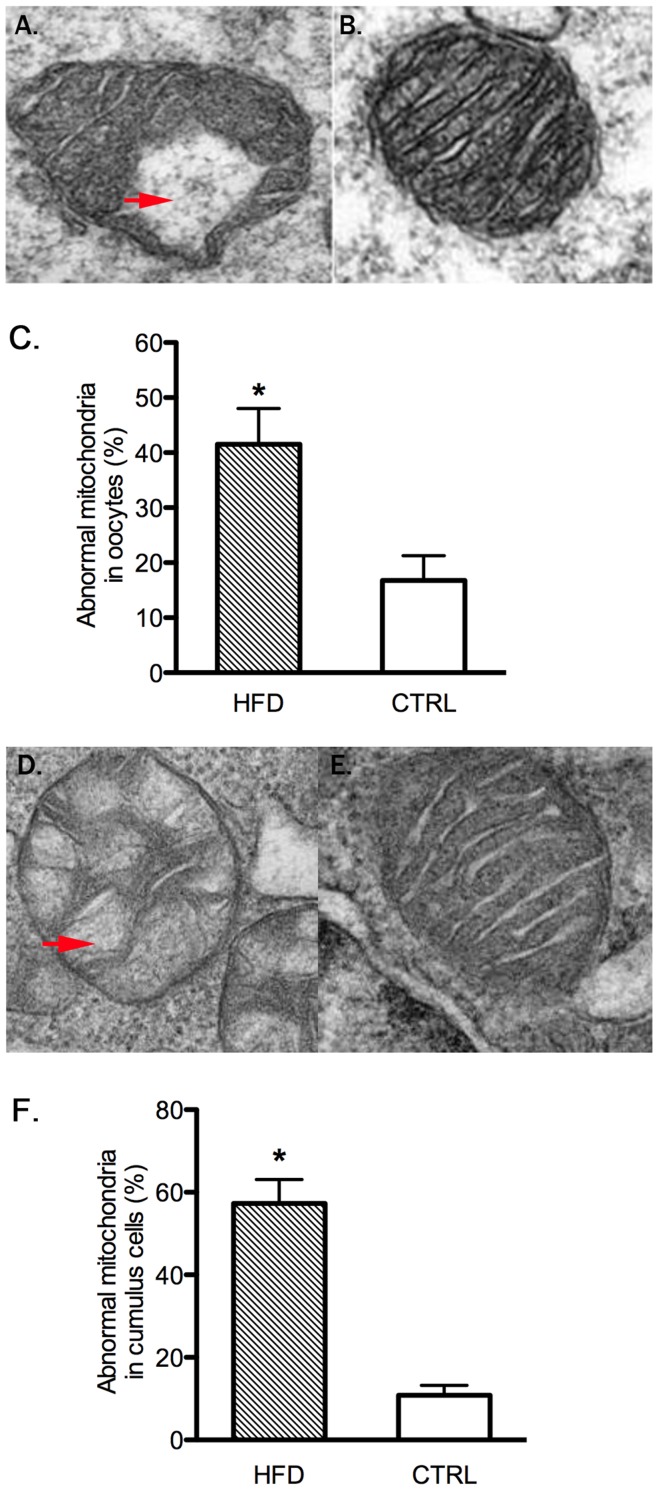
Oocyte and Cumulus cell mitochondrial ultrastructure. Oocyte mitochondria from HFD [A] and Control [B] mice. [C] Histogram shows that oocytes from HFD have higher rates of abnormal mitochondrial morphology. Cumulus cell mitochondria from HFD [D] and control [E] mice. [F] Histogram demonstrating that cumulus cells from HFD have higher rates of abnormal mitochondrial morphology. Arrows indicate the vacuoles in mitochondria.

**Figure 5:**
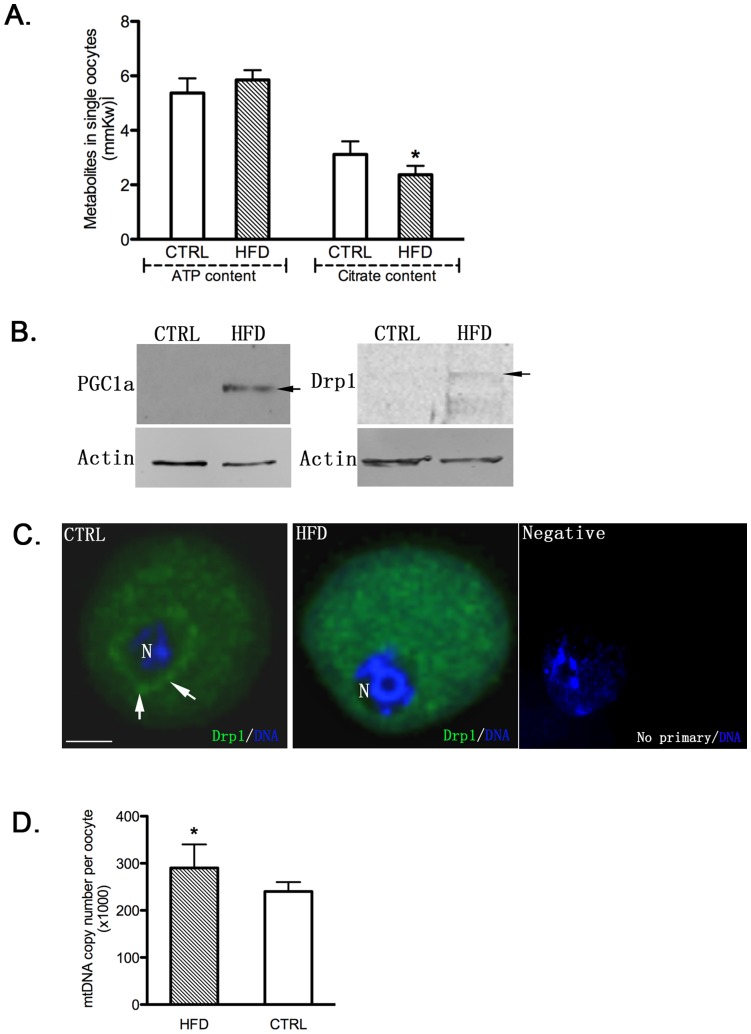
Oocyte Mitochondrial function. [A] Abnormal mitochondrial metabolism in HFD denuded GV oocytes. A. Individual oocytes were assayed for ATP [left] and Citrate [right] levels [mmoles/kg wet weight/oocyte]. Only citrate levels were significantly different in the HFD [black bar] oocytes. *p<0.01. [B] Western immunoblot of denuded oocytes from control vs HFD mice reveal relatively higher protein expression of PGC-1α and Drp1 in the HFD group. [C] Immunofluorescent detection of Drp1 protein in denuded oocyte from Control vs HFD fed mice shows perinuclear staining in control vs diffuse cytoplasmic staining respectively. Negative control with no primary antibody is also included. [D] Mitochondrial DNA copies numbers in denuded oocytes from HFD vs Control mice demonstrates increased mtDNA in the HFD group. *P<0.01.

## Results

### The Obese Uterine Environment is not Required to Produce the Fetal Developmental Defects Observed in High Fat Fed Mice

We have previously shown that mice fed a high fat diet [HFD] prior to and throughout pregnancy display significant growth retardation compared to control fetuses [15]. To determine if an obese uterine environment was required for the observed fetal defects, blastocysts from HFD mothers were transferred to control lean pseudo-pregnant mothers and fetuses analyzed on embryonic day 14.5 [e14.5]. Mice were placed on a diet in which 36% was fat by nutrient content, comparable to the standard Western Diet, for four weeks [26]. We have previously shown that such mice are 19% larger than control-fed mice following the diet before pregnancy [27]. Following superovulation and mating, equivalent numbers of morphologically normal blastocysts from mothers on control vs HFD were transferred into control recipients on normal chow diet. Each experiment was repeated in triplicate for a total of 15 mice in each group, leading to evaluation of 49 embryos/placentas from control mice and 53 embryos/placentas from HFD mice. Overall implantation rates [45.7% versus 55.6%] and pregnancy rates [41.1% versus 50.5%] were not statistically different between the HFD and control groups [data not shown]. At e14.5, embryos and the associated placentas originating from mothers on a high fat diet were significantly smaller by crown-rump length or diameter, respectively, and weighed significantly less than those originating from a control (Figure 1A–B). All embryos were recovered from each mouse, measured and checked by Kolmogorov-Smirnov test for equal, continuous probability distribution. No one female contributed a skewed fraction of the total number of embryos and the distribution by the K-S test was normal.

Necropsies of the e14.5 embryos were randomly performed on fetuses [1 per mother] from 10 different control and 10 different HFD mothers both transferred into control recipients were evaluated. A significant difference in brain development was consistently seen in the embryos resulting from transfer of blastocysts from the HFD into control mice (Figure 1C). Forty percent [4 of 10] displayed structures consistent with reduced ventricular development and an underdeveloped choroid plexus. An additional 20% [2 of 10] had isolated hydrocephaly. No brain abnormalities were seen in any of the 10 control fetuses. All other major organs were unaffected. Together, these results demonstrate that an obese uterine environment is not required for the observed developmental defects.

**Figure 6:**
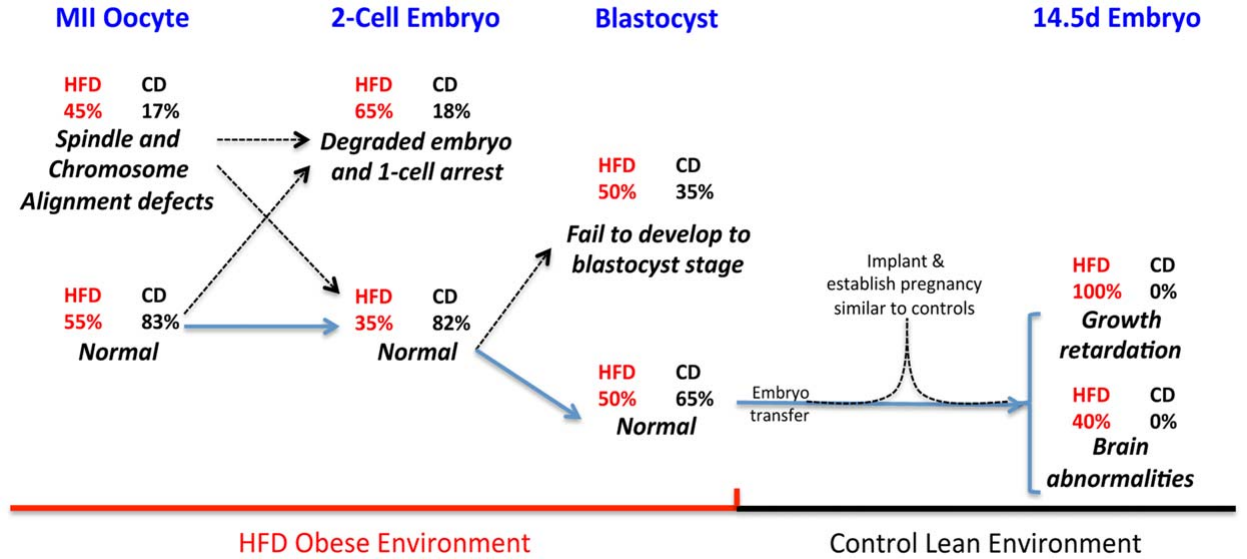
Summary of developmental phenotypes observed in oocytes and embryos from HFD obese mice. A developmental time line is shown, from the ovulated MII oocyte through embryonic day 14.5, with the HFD obese or lean diet/environment indicated below for the relevant stage. The percentages for each phenotype are derived from Figures 1, 2 and 3 and the text and are indicated for the HFD [red] and the control diet [black]. Blue arrows indicate the developmental progression – experimental manipulation while the dashed arrows indicate the inferred contribution to the phenotype. The term “normal” indicates the percent at that stage that appear cytologically normal, although oocytes/embryos at that stage may contribute to abnormal individuals later in the developmental time line.

### Embryos from Mothers Fed a High Fat Diet are Impaired at the Two-cell Stage

To determine whether or not the HFD-induced impairments were evident earlier in development, we collected embryos from HFD and control mice 46 h post-hCG treatment and assessed their morphology (Figure 2). While the total number of embryos from control vs HFD was not different, suggesting that ovulation and fertilization was normal, the morphology and development differed. More than 80% of embryos collected from control mice at 46 h post-hCG have symmetrical two-cell morphology with very few degraded or one cell embryos (Figure 2A and 2B). In contrast, embryos collected from HFD mice included an equivalent number of two-cell [∼35%] and one-cell embryos [∼35%], and a slightly lower proportion of degraded embryos [∼30%] (Figure 2A and 2B). The two-cell embryos were then separated and allowed to progress to the blastocyst stage. The percentage of embryos developing to a blastocyst stage was significantly lower among embryos from the HFD mice vs control [∼50% vs 65%] (Figure 2C). In sum, only ∼20% of the embryos obtained 46 h post-hCG from mothers on the HFD progress to the blastocyst stage, but if they develop to the blastocyst stage will go on to implant and establish pregnancy similar to controls.

### Oocytes from Mothers Fed a High Fat Diet have Spindle and Chromosome Alignment Defects

Our observation that embryos were defective as early as the two-cell stage suggested the possibility of an oocyte maternal effect. Furthermore, we have previously observed a higher incidence of spindle abnormalities and metaphase II [MII] chromosome misalignments in type 1 diabetes models [18]. We therefore evaluated ovulated MII oocytes for spindle and chromosome defects. Oocytes from control mice had typical-barrel shape spindles and well aligned chromosomes on the metaphase plates. Oocytes from mice on the high fat diet presented meiotic defects showing ectopic organizing centers and fragmented/malformed spindles, and chromosomes that were displaced from the metaphase plate (Figure 3A and 3B). Both spindle defects and chromosome misalignment were increased significantly in HFD mice compared to control mice (Figure 3C). Chromosome spreads also showed a higher incidence of aneuploidy in the HFD MII oocytes [data not shown]. Based on the MII oocyte spindle defects and chromosome misalignment, it is likely than a significant proportion of the zygotes will be aneuploidy, which is likely to explain, at least in part, the high frequency of embryos that are abnormal at the two cell stage and fail to reach the blastocyst stage.

### Mitochondrial Abnormalities

The above results indicate that the maternal HFD has profound effects as early as the oocyte stage and may perturb metabolism through impaired mitochondria. To explore this possibility, we first used transmission electron microscopy to examine mitochondrial morphology in GV stage oocytes from both HFD and control mice. Oocyte mitochondria from mice on the high fat diet displayed fewer cristae that were more disarrayed, decreased electron density of the matrix, increased swelling, and more vacuoles (Figure 4A). Normal oocyte development depends on bidirectional communication between the oocyte and the surrounding follicle cells, pathways that rely on normal energy metabolism [28]. We thus assessed mitochondria structure in cumulus cells and found, similar to oocytes, that they exhibited a higher frequency of structural defects including disarrayed cristae, decreased density of the matrix, and increased swelling and vacuoles (Figure 4D).

Given the abnormal morphology of the mitochondria, we reasoned that they would likely not be fully functional. First, employing enzymatic cycling assays [9] of individual oocytes, we found significantly lower citrate levels in oocytes from HFD mice compared to controls, but normal ATP (Figure 5A), suggesting mitochondrial stress without significant compromise of oocyte metabolism [29]. We then examined mitochondrial DNA copy numbers and found that they were significantly higher in the oocytes from the HFD mice than from mice on control diet (Figure 5D), suggesting a compensatory increase due to mitochondrial damage, as previously suggested for type1 diabetic mouse oocytes with abnormal mitochondrial function [18] as well as for mice fed a high fat diet for 6 weeks as described by another group [19]. Data are accumulating which show peroxisome proliferator-activated receptor gamma co-activator [PGC-1α] to be a master regulator of mitochondrial biogenesis in mammals, through powerful induction of the expression of nuclear respiratory factors [30]. Dynamin-related protein 1 [Drp-1], a large GTPase, mediates mitochondrial fission in mammalian cells [31]. Notably, Western blot analysis on GV oocytes from control vs HFD mice revealed increased expression for these two markers, PGC-1α and Drp-1 (Figure 5B). Immunofluorescence also showed not only a significant increase in Drp-1 signal, consistent with the western blot, but also detected a diffuse cytoplasmic pattern in the HFD oocytes whereas the controls exhibited a distinct perinuclear distribution of Drp-1 (Figure 5C). Together, these results suggest that mitochondrial stress induced damages leads to an attempt to compensate through increased mitochondrial biogenesis.

## Discussion

Maternal obesity is well known to have negative impacts on fertility. We have identified a number of reproductive developmental defects that arise from high fat diet induced obesity in the mouse system, which are summarized in Figure 6. Of embryos expected to be at the two-cell stage from obese mothers, ∼65% are abnormal being either degraded or remaining at the 1 cell stage. Of the two-cell stage embryos of normal morphology, only 50% proceed to the blastocyst stage. Thus there is an early embryonic loss of ∼80% in obese mothers, suggesting an oocyte maternal effect. Analysis of oocytes at the MII stage indicates that ∼45% show significant spindle or chromosome misalignment defects, which are likely to generate embryos with massive aneuploidy. However, the ∼45% of MII oocytes with meiotic abnormalities does not fully account for the ∼80% of zygotes that fail to reach the blastocyst stage, suggesting that there is an additional defective process that results in early embryonic developmental abnormalities. The nature of this additional likely oocyte maternal effect awaits further investigation.

Previously we found that mice fed a HFD prior to and throughout pregnancy had fetuses that were growth retarded, with the live-born pups significantly smaller at birth [15]. Here we used blastocyst transfers to demonstrate that growth retardation, as well as brain developmental abnormalities, were not a result of an obese uterine environment. Instead, the defect must occur prior to the blastocyst stage, possibly also as early as the oocyte stage. Importantly the blastocysts used in the transfer assay had normal morphology and implanted and established pregnancy similar to controls, suggesting that the defective processes that leads to the fetal growth retardation and brain abnormalities is distinct from the defects that lead to the early embryonic abnormalities. Thus we propose that the HFD obese maternal environment leads to at least three abnormal reproductive developmental processes which are likely oocyte maternal effects: meiotic aneuploidy that contributes to early embryonic loss; a defect distinct from meiotic aneuploidy that leads to early embryonic loss; and fetal growth retardation and brain developmental abnormalities, which must originate from defects prior to the blastocyst stage. Determining if the fetal abnormalities are due to an oocyte maternal effect or a problem that arises during cleavage divisions will require transfers of one-cell zygotes from HFD mothers into control diet mothers; however this is technically challenging given the high rate of pre-blastocyst lethality. The basis of the fetal abnormalities are not known, although we note that a failure to maintain paternal imprints in the early embryo could result in growth retardation phenotypes.

Obese women commonly deliver macrosomic infants, and thus our model would appear to be contradictory. Two points, however, need to be clarified. First, mice are delivered at a much less mature stage than humans. This has been shown in the development of the immune system, respiratory system as well as neural-sensory system. Thus, birth weight in a mouse may reflect an earlier developmental timepoint than in human neonates. We have found that these smaller fetuses from mothers fed a high fat diet throughout pregnancy catch up within 21 days of birth and exceed their aged match controls, perhaps reflecting the larger size human newborn. Secondly, among women with preexisting severe and morbid obesity, a significant percentile delivers growth retarded or small for gestation age [SGA, birthweight below the 10th percentile] infants [32]. These women are believed to have more central adiposity and the prognosis for these infants is much more grave than the standard obese patients [33], [34]. A recent Dutch study reported an overall incidence of SGA at 18% in obese women, significantly higher than 10% in the general population [35].

The brain abnormalities we observed are especially significant in light of results from several recent human studies suggesting that maternal obesity predisposes their infants to a greater risk of neurodevelopmental delay [36] and atypical neurodevelopment [37], [38] than those born to healthy, lean women. Furthermore, a recent review examining 12 studies that explored the connection between maternal obesity and cognitive, behavioral, and emotional problems in offspring and supported the conclusion that the offspring of obese women may be at increased risk of behavioral and cognitive deficits in childhood, as well as eating disorders in adolescence and psychotic disorders in adulthood [39]. In response to such findings, the Institute of Medicine has highlighted neurodevelopment as an important potential long-term consequence of gestational weight gain that needs further investigation [40]. Our study suggests that preconceptional weight gain adversely affects pregnancy outcomes and fetal development. Further studies into the behavioral phenotypes of these offspring will strengthen this hypothesis. In light of our findings, preconceptional counseling may be indicated as the preferable, earlier target for intervention in obese women desiring pregnancy and healthy outcomes.

We observed that oocytes from mice on a high fat diet displayed abnormal mitochondrial metabolism as assessed by citrate levels in individual denuded oocytes. Oocytes from HFD mice also had significantly higher numbers of abnormal appearing mitochondria, characterized by intra-organelle vacuoles, swollen cristae and less dense matrix as compared to control diet fed mice. Moreover, as has been shown to occur in other mtDNA rich cells such as cardiomyocytes and skeletal muscle, increased reactive oxygen species led to induction of specific transcription factors in order to increase mitochondrial biogenesis, evident by elevated mtDNA copy number and increased accumulation of proteins responsible for mitochondrial biogenesis and fission, consistent with results reported by Igosheva et al [19]. Cumulus cells from the same high fat fed mice also demonstrated abnormal mitochondrial ultrastructure. It has been established in several systems that high fat feeding results in changes in mitochondrial membrane fatty acid composition, and it has been suggested that these changes lead to significant alterations in uncoupling proteins and energy metabolism [41], [42]. By extension, our study would be the first to suggest that altered mitochondrial membrane fatty acid composition may occur in the mammalian oocyte - cumulus complex and that this perturbation can manifest as early as after one month of the diet. It will be interesting to revisit the maternal HFD model to examine redox state in the mitochondria and for how long these metabolic changes persist following fertilization and embryonic development in a non-obese environment.

Skeletal muscle is a major model for study the effects of HFD and diabetes on physiology, cellular and mitochondrial function [43]. Similar to oocytes, the HFD induces abnormal mitochondrial function and ultrastructural abnormalities in skeletal muscle including swelling, disarrayed cristae and reduced matrix [44], [45]. However, unlike the oocyte, in skeletal muscle there is decrease in mitochondria number and decreased expression of mitochondrial gene products, including PGC1α [44]. This distinct mitochondrial biogenesis response to the HFD likely reflects the different biological functions of the two cell types.

Previously, we found disordered spindles and misaligned chromosomes in MII oocytes from mouse models of type 1 diabetes [18], which also display abnormal mitochondrial metabolism and ultrastructure. It is thus tempting to speculate that the deficits in mitochondrial function are the cause of the meiotic spindle and chromosome alignment defects. Although the mechanistic basis might be similar, the proportions of oocytes exhibiting abnormal chromosome alignment, aberrant spindle formation and aneuploidy were much higher in this model of HFD compared to the hyperglycemic type1 diabetic model [18]. This difference may be caused by hormonal or environmental differences, such as the level of oxidative stress, which could be more severe in this HFD model of obesity and type 2 diabetes [46].

Our finding that there are three abnormal reproductive developmental processes in a HFD mouse model of obesity informs future studies on reduced fertility and congenital abnormalities observed in obese woman, which is an increasing proportion of the population. Our proposal that the reproductive developmental defects may arise from an oocyte maternal effect is consistent with IVF studies where the increased pregnancy failure rate in obese women returns to the normal rate if donor oocytes are used instead of autologous oocytes [8]. Our four-week HFD obesity model suggests that the oocyte defects arise in the antral follicles that are recruited in the latest cycle, exposed to the highest effect of the HFD environment. This suggests potential therapeutic routes for reversing the oocyte reproductive obesity effects through diet and exercise.
